# Dynamics and Regulation of Insulin Secretion in Pancreatic Islets from Normal Young Children

**DOI:** 10.1371/journal.pone.0165961

**Published:** 2016-11-02

**Authors:** Jean-Claude Henquin, Myriam Nenquin

**Affiliations:** Unit of Endocrinology and Metabolism, Faculty of Medicine, University of Louvain, Brussels, Belgium; Joslin Diabetes Center, UNITED STATES

## Abstract

Insulin secretion has only exceptionally been investigated in pancreatic islets from healthy young children. It remains unclear whether those islets behave like adult islets despite substantial differences in cellular composition and higher β-cell replication rates. Islets were isolated from 5 infants/toddlers (11–36 month-old) and perifused to characterize their dynamics of insulin secretion when subjected to various stimuli and inhibitors. Their insulin responses were compared to those previously reported for similarly treated adult islets. Qualitatively, infant islets responded like adult islets to stimulation by glucose, tolbutamide, forskolin (to increase cAMP), arginine and the combination of leucine and glutamine, and to inhibition by diazoxide and CaCl_2_ omission. This similarity included the concentration-dependency and biphasic pattern of glucose-induced insulin secretion, the dynamics of the responses to non-glucose stimuli and metabolic amplification of these responses. The insulin content was not different, but fractional insulin secretion rates were lower in infant than adult islets irrespective of the stimulus. However, the stimulation index was similar because basal secretion rates were also lower in infant islets. In conclusion, human β-cells are functionally mature by the age of one year, before expansion of their mass is complete. Their responsiveness (stimulation index) to all stimuli is not smaller than that of adult β-cells. Yet, under basal and stimulated conditions, they secrete smaller proportions of their insulin stores in keeping with smaller in vivo insulin needs during infancy.

## Introduction

Inadequate insulin secretion disrupts glucose homeostasis at all ages, including infancy. Much attention has been paid to the mechanisms causing excessive insulin secretion in congenital hyperinsulinism [1–3] and insufficient secretion in monogenic neonatal diabetes [4, 5], and to possibly predictive β-cell defects in children at risk of type 1 diabetes [6]. In contrast, the secretory function of pancreatic β-cells has rarely been investigated in healthy infants or toddlers, largely for ethical reasons. Compared with islets from adolescents and adults, islets from young children display substantial evolving differences in cellular composition [7–11] and higher rates of β-cell replication [10, 11], but it is unclear how these features impact on their secretory function.

Normal neonates display higher plasma insulin/glucose ratios than infants and children [12, 13], and show larger rises in plasma insulin concentration during iv infusion of amino acids than glucose [14, 15], two characteristics compatible with some “immaturity” of their β-cells. In 1–7 day-old newborns, iv injection of glucose was followed by a rapid increase of plasma insulin in the umbilical vein [16]. In peripheral blood, the amplitude of the rapid insulin response to iv glucose slightly augmented with body weight in children between 1 and 3 y [17], and with age between 4 and 10 y [18, 19], until occurrence of a marked increase at the time of puberty [20, 21]. In pre-pubertal children, the increase in plasma insulin was biphasic during hyperglycemic clamps [20, 22].

Three-to-four decades ago, insulin secretion by the human fetal pancreas has been extensively studied in vitro. The consensus that emerged was that between 14 and 22 weeks of gestation, human fetal β-cells poorly responded to stimulation by glucose alone, but that a response could be unmasked by cAMP-raising agents, and that amino acids and sulfonylureas were more effective than glucose [23–28]. In contrast, in vitro studies of insulin secretion by the pancreas from normal neonates, infants or toddlers are very rare and somewhat contradictory. In islet-like cell clusters from two neonates (2 and 5 weeks) born at term, high glucose induced a rapid release of insulin with little second phase except when theophylline was added to increase cAMP [29]. Islets isolated from one 6-month-old infant responded to high glucose by a biphasic secretion of insulin [30]. Fragments of unaffected pancreas from infants with focal forms of congenital hyperinsulinism were found to secrete insulin in a qualitatively similar way to islets isolated from healthy adult organ donors [31]. Conversely, a recent study of islets isolated from 3 infant organ donors reported poor insulin secretion in response to glucose and KCl, and rapid loss of function compared with adult islets [32]. In this paper, we report a detailed characterization of the dynamics and regulation of insulin secretion by islets isolated from 5 infant and toddler organ donors.

## Methods

During a previous in vitro study of insulin secretion by islets from normal adult donors [33], we received islets isolated from the pancreas of 5 young children (11–36 months of age) referred to the transplantation Unit of the Medical Faculty of the University of Louvain through the Eurotransplant Network. The study was conducted within the framework of programs of basic research and islet transplantation approved by the Ethics Committee of our Institution (UCL-HIA-001, authorization 2001/79) and consent was given by the donors’ parents. The procedures of islet isolation and culture, and the technique of islet perifusion used to characterize insulin secretion were identical to those reported for adult islets [33]. Characteristics of the donors and of their isolated islets are given in Table 1. Although the group of donors includes both infants and toddlers, only the term “infant” will be used subsequently for the sake of simplicity.

**Table 1:** Characteristics of infant donors and of their isolated islets.

Donors					Islets				
Age (mo)	Sex	BW (kg)	Cause of death	Heart-beating	CIT	Purity (%)	Viability (%)	Culture duration	Insulin content (ng/islet)
11	F	8	Child abuse: head trauma	Yes	1h15	90	90	35/60 h	10.1
12	M	9	Pneumococcal meningitis	Yes	1h00	95	95	34/58 h	9.8
22	M	13	Traffic accident	No:3min	4h50	65	85	37 h	12.5
24	F	12	Child abuse: head trauma	Yes	4h45	50	90	32/38 h	18.9
36	M	17	Traffic accident	Yes	2h45	80	86	35 h	17.9

CIT: Cold Ischemia Time

In brief, after culture in RPMI medium containing 5mmol/l glucose for about 38 h, similar portions of each islet preparation were transferred into perifusion chambers and perifused with a bicarbonate-buffered salt-balanced solution supplemented with glucose and test agents as required [33]. Because individual islets were not hand-picked and counted before transfer into chambers, the islet number used in each experiment was estimated by dividing the total amount of received islets (determined with islet purity after dithizone staining of a preparation sample) by the number of chambers [34]. Depending on the donor, 125–335 islets were used per perifusion chamber. To normalize results, islets were recovered from the chambers at the end of experiments and insulin was extracted in acid-ethanol [35]. Insulin was measured in effluent fractions collected every 2-min and in extracts. Fractional insulin secretion rate was then calculated as the percentage of insulin content secreted per minute, which is independent of differences in islet number between experiments [33,34]. Most data are presented as fractional insulin secretion rates (means ± SE). A stimulation index (ratio of stimulated to basal secretion rates) was also calculated in some experiments.

Results in infant islets were compared with those previously obtained in adult islets that were isolated, purified, counted, cultured and eventually perifused in exactly the same way and over the same time period [33]. Characteristics of both groups of islet preparations were similar: purity averaged 76% (50–95) in infants and 68% (30–95) in adults; viability averaged 89% (85–95) in infants and 87% (70–93) in adults; culture duration averaged 38 h (32–60) for infant islets and 46 h (28–76) for adult islets. The dynamics of insulin secretion by adult islets have previously been published [33] and are not reproduced here. However, some results obtained with these adult islets were recalculated to permit comparison of the magnitude of insulin secretion by adult and infant islets. The statistical significance of differences between both groups was assessed by two-tailed unpaired Student’s t-test, with a threshold at P<0.05.

## Results

### Islet insulin content

The insulin content of islets from infants was estimated by summing insulin secreted during experiments and insulin extracted from islets at the end of experiments. Values for the five individual cases are given in Table 1. Their average of 13.8 ± 1.9 ng insulin per islet is not different from the average content of 16 preparations of adult islets studied over the same time period (14.4 ± 1.4 ng) [33].

### Concentration-dependency of glucose-induced insulin secretion

**Fig 1:**
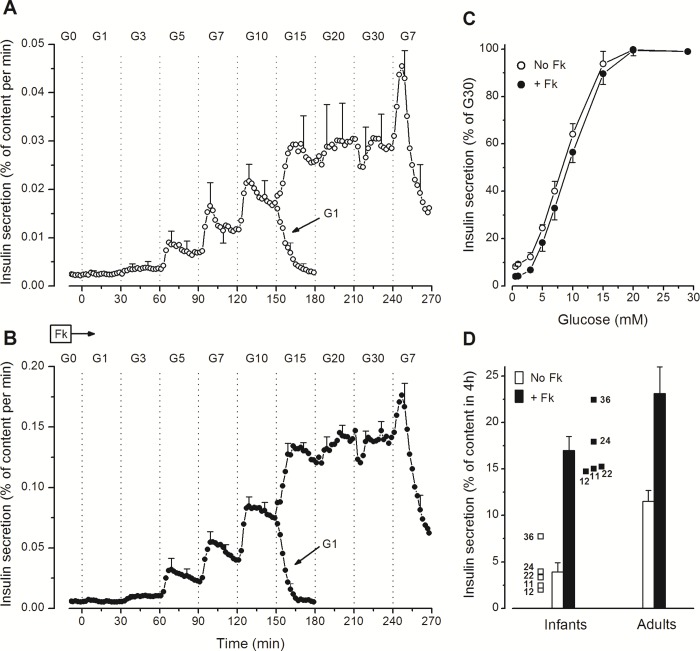
Concentration-dependency of glucose-induced insulin secretion in perifused islets from human infants. (A and B) The concentration of glucose (G in mmol/l) was increased and decreased as indicated, but the islets were not exposed to the whole range of concentrations. One group of islets was perifused in G0 for 60 min before the glucose concentration was increased stepwise to G10 and eventually decreased to G1 at 150 min. Another group from the same preparation was perifused in G7 for 60 min before the glucose concentration was increased stepwise to G30 and eventually decreased to G7. Insulin secretion rates in G7 and G10 from the two series, run in parallel, were similar and therefore averaged to obtain the full dose curve for each of the five islet preparations. Parallel experiments were done in the absence (A) or presence (B) of 1 μmol/l forskolin (Fk) in islets from the five infants. (C) Concentration-dependency curves expressed as percentages of insulin secretion rates in G30. Values are means ± SE for the five infant cases. (D) Total insulin secretion (without and with forskolin) was calculated between 0 and 240 min and is shown for each of the five infant cases identified by their age in months. Columns show means ± SE for the five infant cases and for previously reported results with 8 preparations of adult islets [33].

Stepwise increases in the glucose concentration progressively increased insulin secretion in infant islets (Fig 1A and 1B). Alone, 1mmol/l glucose (G1) was ineffective (compared to G0), G3 doubled insulin secretion in two preparations (11 and 36 months), and G5 was stimulatory in all preparations (Fig 1A). The secretion rate then kept increasing up to G15 with hardly any further change above. Switching from G20 to G30 transiently decreased insulin secretion in 3 out of 5 preparations. When the glucose concentration was lowered from G10 to G1 (at 150 min), insulin secretion rapidly returned to basal rates. In contrast, lowering from G30 to G7 (at 240 min) was initially followed by a transient increase (off-response) (Fig 1A). The pattern of these changes in insulin secretion, including the unexplained off-response, was similar in the 5 islet preparations (S1 Fig) and is virtually identical to that previously observed in adult islets [33]. When the perifusion medium was supplemented with 1 μmol/l forskolin to increase islet cAMP levels, qualitatively similar but quantitatively larger responses were observed (Fig 1B). These experiments thus showed that the glucose-dependency of insulin secretion by islets from human infants was similar without or with forskolin (Fig 1C), with a threshold at G3-G4, half-maximal stimulation between G7 and G10, and maximum stimulation slightly above G15.

Total insulin secretion over the 240 min of stimulation by glucose alone or with forskolin is shown for each of the five cases in Fig 1D. Compared with adult islets previously tested with a similar protocol [33], infant islets secreted a smaller proportion of their insulin reserves when stimulated with glucose alone (3.9 ± 1.0% versus 11.5 ±1.2% in 4h, P< 0.01), but the difference was markedly attenuated (17.0 ± 1.5% versus 23.4 ± 2.9% in 4h, NS) in the presence of forskolin. Indeed, raising cAMP produced a greater amplification of the insulin response to glucose in infant (4.4-fold) than adult islets (2.0-fold).

### Dynamics of insulin secretion

**Fig 2:**
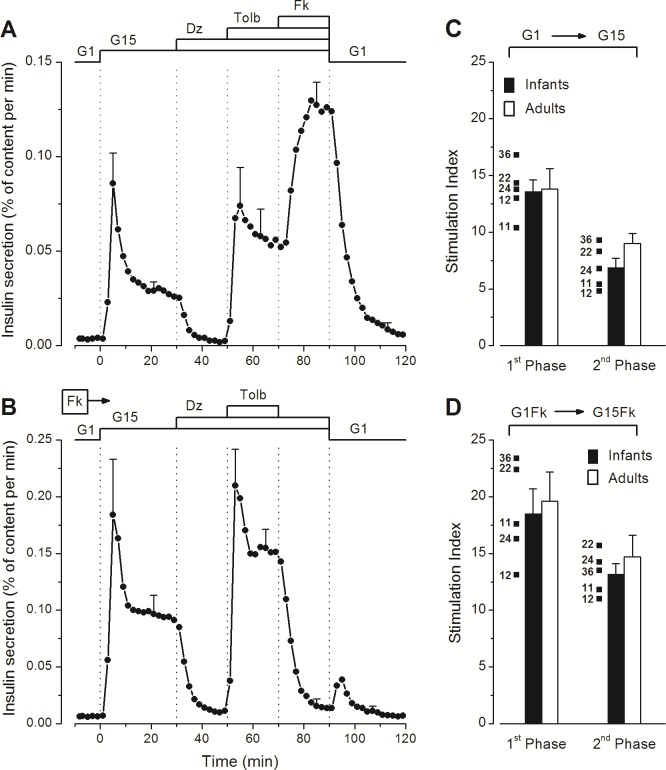
Dynamics of glucose-induced insulin secretion in perifused islets from human infants. The experiments also tested the effects of drugs opening (diazoxide) or closing (tolbutamide) K_ATP_ channels and of an increase in islet cAMP by forskolin. (A) The concentration of glucose was changed between 1 and 15 mmol/l (G1, G15), and diazoxide (Dz, 100 μmol/l), tolbutamide (Tolb, 100 μmol/l), and forskolin (Fk, 1 μmol/l) were added and withdrawn as indicated. (B) The whole experiment was performed in the presence of 1 μmol/l forskolin. Values are means ± SE for the five infant cases. (C and D) Glucose-induced insulin secretion was expressed as a stimulation index (ratio G15/G1) during first phase (2–10 min) and second phase (20–30 min) of the response. Mean and individual values for islets from the five infants (identified by age in months) are compared with mean values for 14–16 islet preparations from normal adults [33].

Stimulation of infant islets by a rapid switch from G1 to G15 induced biphasic insulin secretion (Fig 2A). Opening K_ATP_ channels with diazoxide abolished insulin secretion, whereas subsequent closure of the channels with tolbutamide completely reversed the inhibition, and addition of forskolin strongly amplified the secretory response. Complete reversibility of the stimulation was observed on returning to G1 alone (Fig 2A). This pattern of insulin secretion was observed in all five preparations, independently of donor age (S2 Fig). The presence of forskolin during the whole experiment augmented the amplitude of first phase (~2-fold) and second phase (~3-fold) of the response to G15 without changing the time course or altering the inhibition by diazoxide and stimulation by tolbutamide. Simultaneous withdrawal of diazoxide and return to G1 was followed by a transient off-response in 3/5 preparations (Fig 2B) (S2 Fig).

The dynamics of insulin secretion in infant islets (Fig 2A and 2B) were thus similar to those previously reported for adult islets, but fractional rates of secretion were lower [33]. Total insulin secreted between 0 and 30 min averaged 1.10 ± 0.16% versus 2.66 ± 0.31% in G15 alone (P<0.01), and 3.16 ± 0.54% versus 4.40 ± 0.48% (NS) in G15 with forskolin. However, the relative amplitude of the stimulation by G15 (stimulation index) was not different in infant and adult islets during either first or second phase of the response (Fig 2C). This similarity of stimulation index in face of lower fractional rates of secretion in stimulated infant islets is explained by lower basal secretion rates in G1.

### Stimulation by amino acids and metabolic amplification of insulin secretion

**Fig 3:**
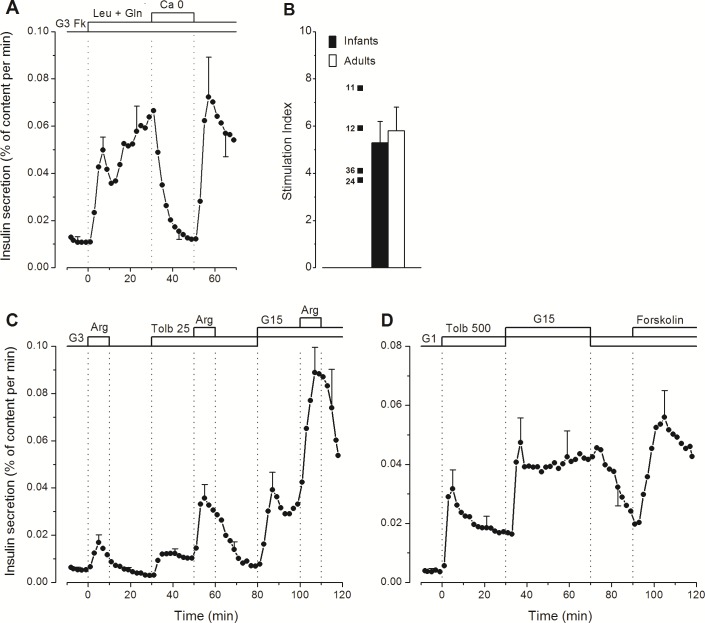
Effects of amino acids and tolbutamide on insulin secretion in perifused islets from human infants. (A) The experiments were done in the presence of 3 mmol/l glucose (G3) and 1 μmol/l forskolin (Fk) throughout. Leucine and glutamine (5 mmol/l each) were added at 0 min. Between 30 and 50 min, CaCl_2_ was omitted and 100 μmol/l EGTA was added. (B) Insulin secretion induced by leucine + glutamine was expressed as a stimulation index for the whole response (0–30 min). Mean and individual values for islets from four infants (identified by age in months) are compared with mean values for 5 islet preparations from normal adults [33]. (C) Three pulses of 10 mmol/l arginine (Arg) were applied in G3 alone, G3 + 25 μmol/l tolbutamide (Tolb 25), or G15 + Tolb 25. (D) Islets were fully depolarized by 500 μmol/l tolbutamide (Tolb 500) in 1 mmol/l glucose (G1). The glucose concentration was then increased to 15 mmol/l (G15) between 30 and 70 min, and forskolin (1 μmol/l) was eventually added to G1 (in 3/5 cases only). Values are means ± SE for islet preparations from 4 (A, B and C) or 5 infants (D).

Addition of a mixture of leucine and glutamine to a medium containing G3 and forskolin induced biphasic insulin secretion, which was reversibly abolished by omission of CaCl_2_ from the medium (Fig 3A) (S3 Fig). The dynamics of the changes were the same as in adult islets [33] but, again, total insulin secretion over 30 min of stimulation was lower (1.48 ± 0.22% versus 3.46 ± 0.69%, P<0.05). Yet, the stimulation index was similar because of lower basal secretion rates in infant islets (Fig 3B).

A 10-min pulse of arginine (10 mmol/l) slightly increased insulin secretion in infant islets perifused in G3 (Fig 3C). Tolbutamide (25 μmol/l) augmented (~3-fold) the secretion rate in G3 and amplified (~3-fold) the response to arginine, which became very large in the presence of G15. These amplifications of arginine-induced insulin secretion involve both augmentation of cytosolic Ca^2+^ (tolbutamide) and augmentation of the response to Ca^2+^ (glucose) [36]. They are qualitatively indistinguishable from those occurring in adult islets [33]. However, fractional secretion rates were lower in infant islets; for example, total insulin secreted during stimulation with tolbutamide and arginine in G3 (30 to 80 min) averaged 0.80 ± 0.11% versus 1.90 ± 0.24% in adult islets, P<0.01).

Metabolic amplification of insulin secretion can be evidenced by increasing the concentration of glucose when all K_ATP_ channels in β-cells are closed by a high concentration of tolbutamide [37]. As shown in Fig 3D, 500 μmol/l tolbutamide rapidly increased insulin secretion in G1, and a subsequent switch to G15 more than doubled the secretion rate in a reversible manner. Addition of forskolin at the end of the experiment was also able to amplify insulin secretion (Fig 3D). The pattern of the response to tolbutamide and of its amplification by high glucose was similar to that in adult islets [33], but total insulin secretion induced by tolbutamide in G1 (0 to 30 min) was smaller (0.63 ± 0.15% versus 2.12 ± 0.40% in adult islets, P<0.01).

## Discussion

In vitro studies of insulin secretion by β-cells from normal young children are extremely rare owing to the exceptional availability of the necessary material. This rarity at least partly originates from the idea that children islets are suboptimal for transplantation and hence infrequently isolated from very young organ donors [30].

Our study shows that the characteristics of insulin secretion by islets from normal infants are similar to those previously defined in similarly treated islets from normal adults [33]. Qualitatively, infant islets responded like adult islets to stimulation by glucose, tolbutamide, forskolin, arginine and the combination of leucine and glutamine, and to inhibition by diazoxide and extracellular CaCl_2_ omission. This similarity included the biphasic pattern of glucose-induced insulin secretion and the dynamics of the responses to other stimuli. Amplification of insulin secretion by glucose when all K_ATP_ channels are closed by a sulfonylurea was operative as in adult islets, as was amplification of arginine-induced insulin secretion by tolbutamide and by glucose [33].

Morphometric studies of the intact pancreas have shown that the average diameter of islets is smaller in children than adults [10, 11], but we have not verified whether a similar difference characterized the preparations of isolated islets that we studied. Anyhow, it is unlikely that the insulin content of infant islets was found similar to that of adult islets because of our use of larger islets from infants than adults. Moreover, insulin secretion rates were expressed relative to islet insulin content, which is independent of differences in islet size and number. Quantitative differences were found between insulin responses of infant and adult islets, but their detection depended on the way of calculation. Although islet insulin content was not different, fractional insulin secretion rates were lower in infant than adult islets irrespective of the stimulus. Yet, these differences did not reflect poor responsiveness of infant β-cells because basal fractional insulin secretion rates were also lower, so that fold-changes above baseline (stimulation index) were similar to those in adult islets. That means that infant β-cells secrete a lower proportion of their insulin content than adult islets under basal and stimulated conditions. It is tempting to relate that peculiarity to the low needs of insulin when, well before puberty, target tissues are still highly sensitive to insulin action. However, it is uncertain whether the elusive mechanisms permitting β-cells to adapt their secretory function to insulin sensitivity in vivo are long-lasting and still operative in vitro after 36h of islet culture. During growth and body weight increase, two factors may underlie the augmentation of insulin production in vivo: an increase in the secretory response of individual β-cells and an expansion of β-cell mass. This expansion starts soon after birth [9], continues up to ~5 years of age [10] and reaches ~4-5-fold according to estimations [10] and direct measurements [7]. Our observations of a normal secretory function in infant islets indicate that the relatively small proportion (<2%) of proliferating β-cells in these islets [10, 11, 38, 39] has little functional influence on the bulk of other β-cells.

Many β-cells are scattered single or form small clusters in the pancreas of neonates, but most of them reside in well-structured islets after 6 months of age [7, 10, 11, 40]. Yet, infant and adult islets still differ in their cellular composition. Whereas the percentage of β-cells is similar, somatostatin cells are relatively more numerous than glucagon cells during infancy, and the proportions are reversed in adulthood [7, 9, 11]. These changes in islet cell proportions during infancy may impact on β-cell function through paracrine mechanisms [41, 42]. Fractional insulin secretion rates were 2.5 to 3-fold lower in infant than adult islets when glucose was used alone, but only 1.4-fold lower (non-significant) when glucose was combined with forskolin to increase β-cell cAMP. These observations indirectly suggest that the lower responses observed in the absence of forskolin might be due to lower basal levels of cAMP in infant β-cells. If direct measurements confirmed the hypothesis, the smaller proportion of glucagon cells and greater proportion of somatostatin cells in infant than adult islets would be a plausible mechanistic explanation. Whereas low cAMP production, a characteristic of fetal β-cells [27, 28], may extend to the first years of postnatal life, no specific anomaly in the action of glucose was detected. The difference in fractional insulin secretion rates between infant and adult islets was not greater during glucose stimulation than during stimulation with tolbutamide, either alone or combined with arginine. In addition, the sigmoidal concentration-dependency curve of glucose-induced insulin secretion in infant islets was close to that in adult islets [33, 34], with a threshold around G3 and maximum stimulation at or just above G15. Only the half-maximally effective glucose concentration was slightly higher in infant than adult islets (G 7–8.5 versus G 6.5–7). These adult-type responses to glucose indirectly indicate that metabolism of the sugar is also controlled by glucokinase rather than hexokinase-I [28, 43, 44] in infant β-cells.

One recent study compared in vitro β-cell function in islets from adults and from 3 infants (2.5, 4 and 19 months of age) [32]. Using hand-picked islets after 24-48h of culture, the authors reported that infant islets contained very low amounts of insulin (10% of adults) and poorly secreted it upon perifusion with high glucose or KCl (reduced stimulation index). They attributed these defects to insufficient production of metabolic signals (lower ATP/ADP and NADPH/NADP ratios) and reduced availability of exocytotic proteins. They also observed that, unlike adult islets, infant islets rapidly lost their functional phenotype during culture [32]. It is unclear why these results so markedly contrast with the excellent functioning of our five preparations of infant islets. Good stimulation of insulin secretion by glucose was also observed in static incubations of islets from young donors (0.5–17 year-old) [30]. We previously characterized the defects of insulin secretion by β-cells from infants (2–11 months) with congenital hyperinsulinism secondary to inactivating mutations in one of the subunits of K_ATP_ channels [31]. In the focal form of the disease, the lesion was surgically resected with a surrounding rim of unaffected pancreas, which was used as control tissue. Although the experiments were done with suboptimal preparations (digested fragments of pancreas), all changes in insulin secretion measured in control tissue [31] were qualitatively similar to those we report here with isolated islets. There was however one quantitative difference: both basal and stimulated fractional rates of insulin secretion were lower in isolated islets than fragments, probably because fewer β-cells were damaged in isolated islets.

In conclusion, islets isolated from 11–36 month-old children secrete insulin virtually like adult islets when tested in vitro. The only significant difference is quantitative, not qualitative. Whereas the dynamics of their responses to an array of stimuli and inhibitors are similar, infant islets consistently secrete lower proportions of their insulin stores than adult islets, a difference that is attenuated by cAMP. Yet, because unstimulated secretion rates are also lower, the amplitude of their responses above baseline (stimulation index) is not reduced. Human β-cells therefore have reached functional maturity by the age of 1 year, before expansion of their mass is complete. Closer timing of postnatal β-cell maturation and elucidation of its mechanisms will require studies of islets from infants between birth and the age of 1 year, particularly around weaning [45]. Ideally, functional and genetic approaches should be combined to determine whether increased expression or silencing of the same genes as in rodent β-cells [45–47] impact on the secretory process. The endeavor will be challenging owing to the rarity of infant donors, but should prove valuable for the numerous laboratories currently attempting to derive well-functioning β-cells from human pluripotent stem cells.

## Supporting Information

S1 Fig Concentration-dependency of glucose-induced insulin secretion in perifused islets from human infants. Individual responses of the five preparations of infant islets whose mean response is shown in Fig 1.
(PDF)

S2 Fig Dynamics of glucose-induced insulin secretion in perifused islets from human infants. Individual responses of the five preparations of infant islets whose mean response is shown in Fig 2.
(PDF)

S3 Fig Effects of amino acids and tolbutamide on insulin secretion in perifused islets from human infants. Individual responses of the five preparations of infant islets whose mean response is shown in Fig 3.
(PDF)
