# Supplementary figures and images for: Dynamics and Regulation of Insulin Secretion in Pancreatic Islets from Normal Young Children
Source: PLoS One. 2016 Nov 2;11(11):e0165961. doi: 10.1371/journal.pone.0165961 (PMC5091846; doi:10.1371/journal.pone.0165961)

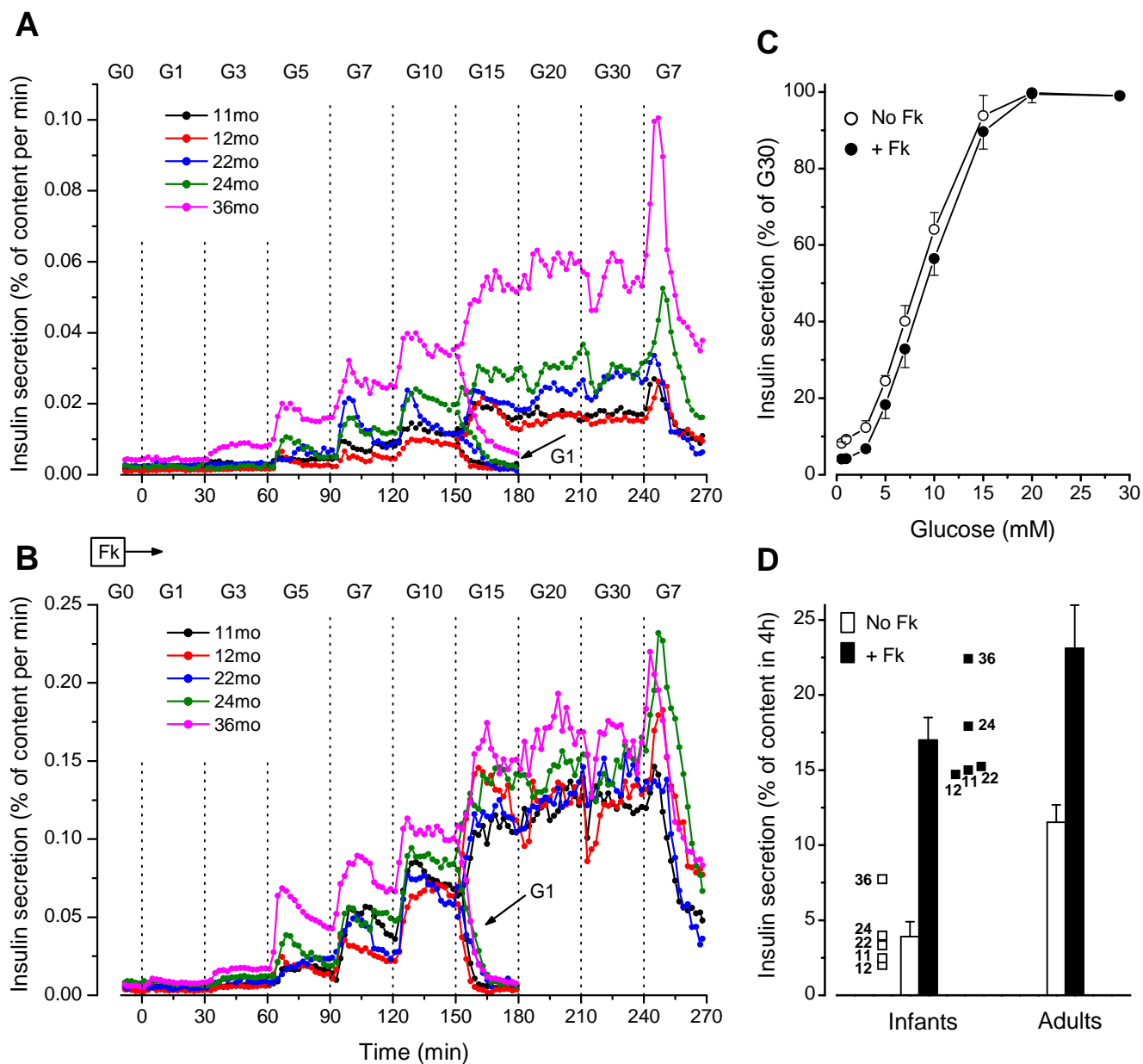

S 1 Fig

Supplement: S1 Fig — Individual responses of the five preparations of infant islets whose mean response is shown in Fig 1. (PDF) [file pone.0165961.s001.pdf]

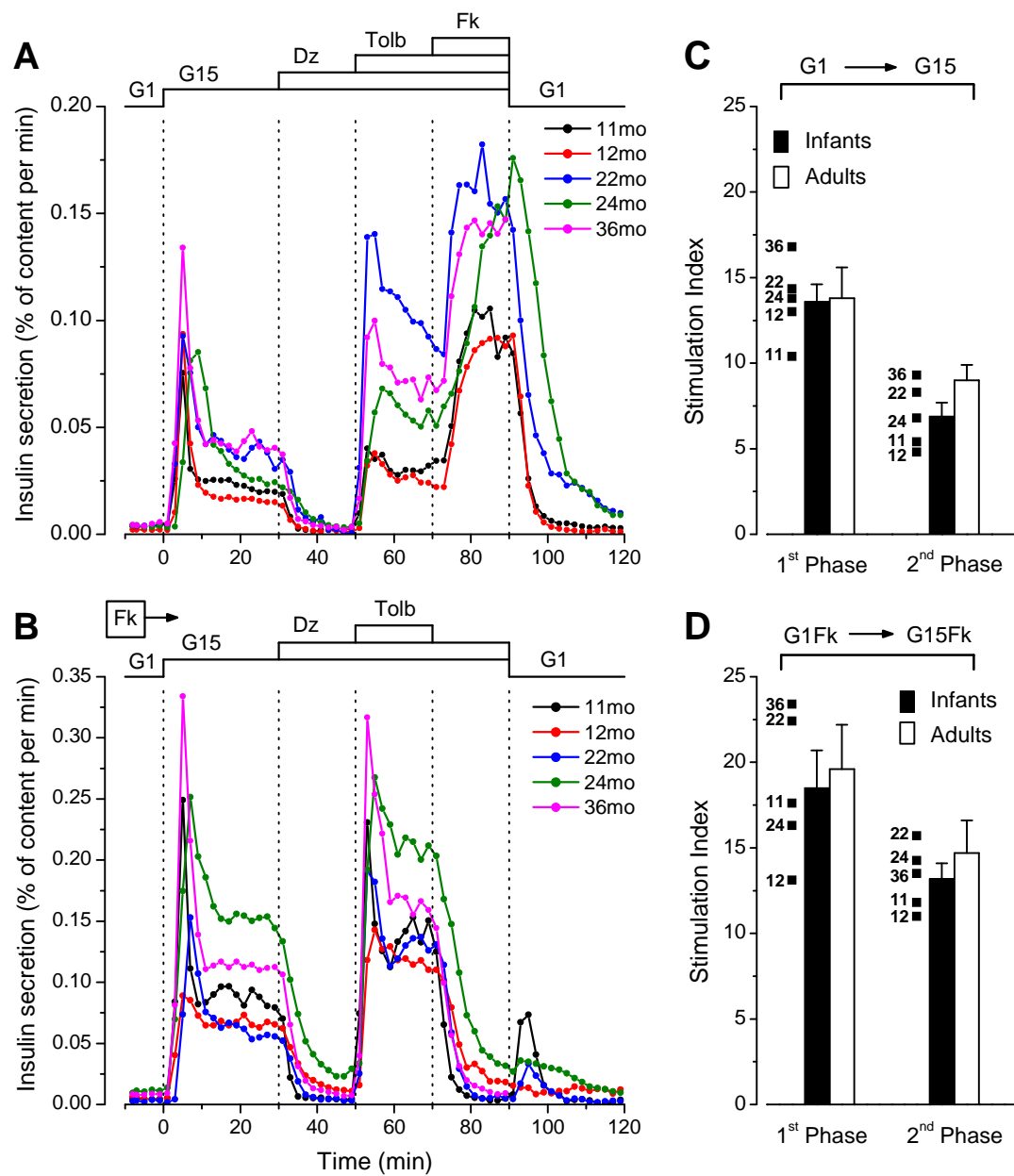

S 2 Fig

Supplement: S2 Fig — Individual responses of the five preparations of infant islets whose mean response is shown in Fig 2. (PDF) [file pone.0165961.s002.pdf]

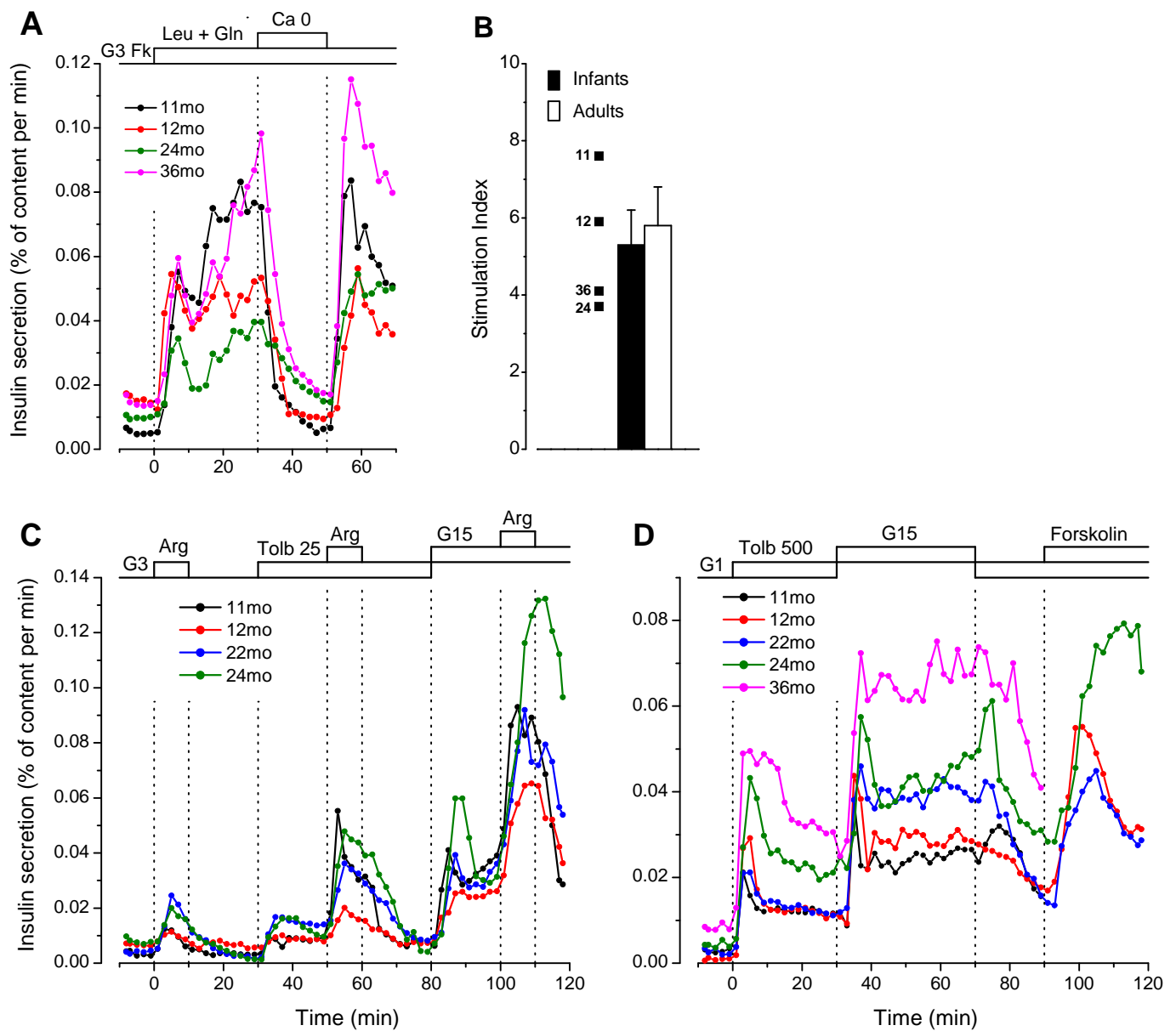

S 3 Fig

Supplement: S3 Fig — Individual responses of the five preparations of infant islets whose mean response is shown in Fig 3. (PDF) [file pone.0165961.s003.pdf]
